# Multimorbidity prevalence and patterns and their associations with health literacy among chronic kidney disease patients

**DOI:** 10.1007/s40620-021-01229-1

**Published:** 2022-01-05

**Authors:** M. S. Gurgel do Amaral, S. A. Reijneveld, L. M. G. Meems, J. Almansa, G. J. Navis, A. F. de Winter

**Affiliations:** 1 Department of Health Sciences, Community and Occupational Medicine, University of Groningen, University Medical Center Groningen, Hanzeplein 1, Building 3217, room 617, 9713GZ Groningen, The Netherlands; 2 Department of Cardiology, University of Groningen, University Medical Center Groningen, Groningen, The Netherlands; 3 Department of Nephrology, University of Groningen, University Medical Center Groningen, Groningen, The Netherlands

**Keywords:** Health literacy, Multimorbidity, Patterns, Chronic kidney disease, Prevention

## Abstract

**Background:**

Health literacy is the ability to deal with information related to one’s health. Patients with low health literacy have poor disease-management skills for chronic diseases, such as chronic kidney disease (CKD). This could influence the number and combination of their diseases.

**Methods:**

We included adult patients with CKD stages 1–5 from the Lifelines Study (n = 2,742). We assessed the association between low health literacy and the number and patterns of comorbidities, considering them globally and stratified by age and sex, using multinomial logistic regression and latent class analysis, respectively.

**Results:**

Low health literacy was associated with a higher number of comorbidities in the crude models, and after adjustment for age, sex, eGFR, smoking, and BMI. In the crude model, the OR for low health literacy increased from 1.71 (1.25–2.33) for two comorbidities to 2.71 (2.00–3.68) for four comorbidities. In the fully-adjusted model, the associations remained significant with a maximum OR of 1.70 (1.16–2.49) for four comorbidities. The patterns of multimorbidity were similar for low and adequate health literacy, overall and by sex, bur tended to be different for patients older than 65. Older patients with low health literacy had higher comorbidity prevalence and a relatively greater share of cardiovascular, psychiatric, and central nervous system diseases.

**Conclusions:**

Among CKD patients, low health literacy is associated with more multimorbidity. Health literacy is not associated with patterns of multimorbidity in younger patients, but a difference was observed in older ones. Improving low health literacy could be an intervention efficient also in decreasing multimorbidity in CKD patients.

**Graphical abstract:**

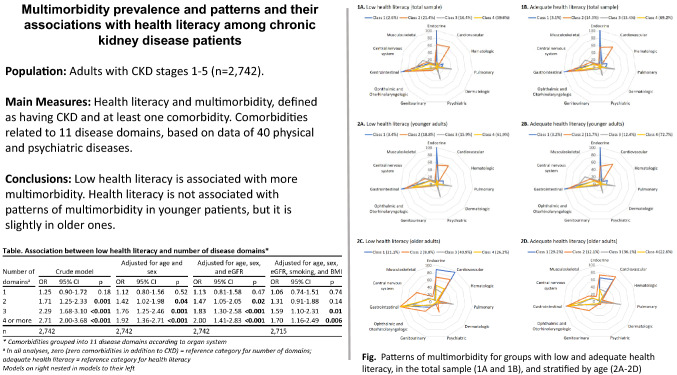

**Supplementary Information:**

The online version contains supplementary material available at 10.1007/s40620-021-01229-1.

## Introduction

Multimorbidity, the co-occurrence of two or more chronic diseases, is a condition that affects up to 95% of patients with chronic kidney disease (CKD) [1]. The term multimorbidity, however, does not specify the disease types and distribution, which are more informative of the impact these diseases have on people’s health. Social determinants of health, such as health literacy [2], may help to determine the patterns of disease distribution in multimorbid patients with CKD, thereby supporting the development of strategies to approach these patients more efficiently.

Multimorbidity is a major challenge for patients with CKD [1], and a more comprehensive understanding of this phenomenon is needed to adapt healthcare systems to their needs [3]. Among CKD patients, multimorbidity is the single highest independent predictor of all-cause mortality [4], and it is an even more immediate concern than kidney function in the management of CKD, particularly during asymptomatic stages [5]. However, current healthcare systems and medical guidelines are generally focused on a single disease, resulting in fragmented and inefficient care, which fails to meet the challenges of multimorbidity [6]. To support the shift of healthcare systems to a more comprehensive model, we need to better understand the factors that determine the number and distribution of diseases in multimorbid patients [7].

Low health literacy is an important determinant of health due to its negative effects on chronic diseases [2], and it may be one of the determinants of multimorbidity among CKD patients. Health literacy is defined as the degree to which individuals have the capacity to obtain, process, and understand basic health information and services needed to make appropriate health decisions [8]. Low health literacy is associated with CKD development and complications [9, 10], mainly because individuals with low health literacy lack the self-management skills required for adequate CKD control [2, 11]. This lack of skills may be especially problematic when the patient presents multimorbidity, since the co-occurrence of other diseases adds to the burden of CKD, making it more difficult to manage [12].

Low health literacy may predict not only the presence of multimorbidity but also its patterns, i.e., the combination of co-occurring diseases. Multimorbidity is more complicated than just a sum of diseases, and the need to understand its patterns is well recognized [13]. The impacts of multimorbidity on CKD patients may vary according to the groups of diseases that occur together [14]. Recent research has identified some determinants of multimorbidity patterns, such as age and sex [15], but no study has directly addressed the association between these patterns and low health literacy. A better understanding of these associations may help to optimize care for CKD patients with low health literacy.

This study aims to assess the association between health literacy and the prevalence and patterns of multimorbidity in CKD patients, overall and divided by age and sex.

## Methods

### Sample and procedures

The sample for this cross-sectional study was derived from the baseline assessment of the Lifelines study. Lifelines is a multi-disciplinary prospective population-based cohort study which examines, in a unique three-generation design, the health and health-related behaviors of 167,729 people living in the north of the Netherlands. It employs a broad range of investigative procedures to assess the biomedical, socio–demographic, behavioral, physical, and psychological factors which contribute to the health and disease of the general population, with a special focus on multimorbidity and complex genetics.

Baseline data collection for adults was performed between November 2006 and December 2013 (*n* = 152,737). Prior to their first visit to the Lifelines outpatient clinic, all participants were asked to complete a self-administered questionnaire, requiring them to provide information pertaining to their medical history, current diseases, use of medication, and health behaviors. Participants were also instructed to bring in a detailed list with information about their latest used medications. During their first visit to the clinic, all participants underwent clinical examination, followed by blood and urine collection, electrocardiography, and spirometry. In addition, current psychiatric disorders (depressive disorder and anxiety disorders) were assessed with a brief standardized diagnostic interview: The Mini International Neuropsychiatric Interview (MINI) 5.0.0. Afterward, participants answered the health literacy questionnaire. More detailed information about recruitment and data collection can be found elsewhere [16]. The Lifelines Cohort Study was conducted in accordance with the principles of the Declaration of Helsinki and the research code of the University Medical Center Groningen [16].

Participants were eligible for our study if they were 18 or older, had completed the health literacy questionnaire, and could be classified as having CKD based on their albuminuria and serum creatinine levels. Exclusion criteria for our study included pregnancy and a history of kidney transplantation, due to the changes in renal function and prognosis that these conditions entail. The eligible sample comprised 2,742 CKD patients, with a mean follow-up time of 4.2 years (standard deviation 1.2 years).

### Measurements

#### Health literacy

Health literacy was measured using self-reported answers to the three validated questions from Chew et al. [17].

1. How often do you have trouble understanding your medical situation because you have difficulty with the written information?
2. How sure are you of yourself when you fill out medical forms?
3. How often does someone help you with reading information materials from the hospital or another healthcare provider?

Participants answered these questions on a Likert scale ranging from 1 (*Never/not at all)* to 5 *(Always/Very)*. We reversed the scores of the first and third questions and then added up the scores of all questions, resulting in a health literacy scale ranging from 3 to 15. This was dichotomized as low (3–12) versus adequate health literacy (13 and above). This cut-off point was also used in previous studies using the same database, and leads to percentages of low and adequate health literacy comparable to those found in large-scale health literacy surveys in the Netherlands [18].

#### Definition of chronic kidney disease (CKD)

In accordance with the latest guidelines [19], to be classified as having CKD a participant needed to meet at least one of the following criteria:

1. Estimated glomerular filtration rate (eGFR) lower than 60 ml/min/1.73m^2^;
2. Albuminuria assessed by 24 h urine ≥ 30 mg/24 h or assessed by albumin-to-creatinine ratio in morning urine ≥ 3 mg/mmol.

The eGFR was calculated with the CKD-EPI formula, which uses values of serum creatinine, age, and sex. Creatinine levels were assessed with serum laboratory tests performed on fasting blood samples drawn from participants at one of the Lifelines research sites. On the same day, participants had to hand in urine samples collected at home. In the sample of adults who completed the health literacy questionnaire, 54% had no values of albuminuria, and only their eGFR was used in the definition of CKD.

#### Comorbidities and the definition of multimorbidity

Forty comorbidities, i.e., single chronic diseases accompanying CKD, were scored according to the 10th edition of the *International Statistical Classification of Diseases and Related Health Problems (ICD-10),* using a combination of data from the questionnaires, medication list, clinical examination, laboratory assessment, electrocardiography, and spirometry. The combination of objective and subjective methods helped circumvent disease misclassification. Individuals had to meet at least one of the diagnosis conditions to be classified as *affected* by the corresponding comorbidity (for a detailed list of the classification criteria, see Supplementary Appendix I). The comorbidities were then clustered into 11 different disease domains, as previously reported [20]: gastrointestinal, cardiovascular, endocrine, pulmonary, central nervous system, ophthalmic and otorhinolaryngologic, psychiatric, musculoskeletal, hematologic, genitourinary, and dermatologic. A disease domain was considered as *affected* when at least one comorbidity was present within that disease domain. This clustering was made because individuals within a domain are likely to be treated by professionals of the same medical specialty, and with similar medications, therefore bearing a similar burden. Cancer was not included because different types of cancer have different physiopathology, risk factors, treatments, prognosis, and could be part of any of the aforementioned disease domains. Therefore, the use of cancer as overall diagnosis would hinder the precision of our comorbidity classification. The disease domains were posteriorly categorized into five groups: 0, 1, 2, 3, and 4 or more disease domains (in addition to CKD). Multimorbidity was present when, simultaneous with CKD, at least one other disease domain was affected.

#### Other variables

In a questionnaire, participants reported sex, age, smoking, educational level, and monthly household income. We calculated BMI using height and weight measured by trained technicians at one of the Lifelines research sites. We defined participants as smokers if they reported any smoking in the previous month [y/n]. We measured educational level using an 8-item ordinal scale (from *No education* to *University education*), and subsequently categorized the answers as: low (no education or complete primary education), intermediate (complete secondary education), and high (higher vocational or university education). We measured monthly household income using an 8-item ordinal scale (from *less than 750 euros* to *more than 3500 euros*), and *I do not know* or *I would rather not answer this question*. The answers were clustered into four categories: *less than 1000 euros*, *1000* to *3000 euros*, *more than 3000 euros*, and *information not given*.

### Analysis

First, we calculated descriptive statistics and evaluated differences between the low- and adequate-health-literacy groups using Pearson’s chi-square tests, independent samples t-tests, or Mann–Whitney tests. Second, we assessed the association between health literacy and the categorized number of disease domains, using multinomial logistic regression. The regression was adjusted for age, sex, eGFR, smoking, and BMI. Age was centralized and included as a confounder with its quadratic polynomial, to allow optimal fitting to the data. The eGFR was dichotomized into higher or lower than 60 ml/min/1.73m^2^. The results of all the models were considered statistically significant if p < 0.05. Results referring to groups with fewer than ten individuals were reported as < *10* (or equivalent percentage), in accordance with the Lifelines requirements for publication to avoid the identification of participants. Subgroup analyses per CKD stage were not possible due to the small power yielded by the subsamples. Third, we assessed differences in patterns of multimorbidity by health literacy, using latent class analysis to define groups of disease domains. Latent class analysis is a model-based approach used to identify homogeneous groups within a heterogeneous population. Individuals in the same class share the same disease probability profile. We examined models with two to eight classes and selected the final model based on the lowest Bayesian information criterion (BIC), the lowest Akaike information criterion (AIC), and the clinical interpretability of the resulting classes. We performed this analysis separately for participants with low and adequate health literacy in the total sample. All analyses were then stratified by sex and age (< 65 and >  = 65), to account for the known association of these variables with health literacy and multimorbidity [21, 22]. The analyses were conducted using *IBM SPSS Statistics* version 25, *Stata* version 16, and *R* version 3.5.2 (package *poLCA*) for Windows.

## Results

### Sample characteristics

Multimorbidity was present in 57% of the total Lifelines sample and 83.3% of the CKD patients. In the total Lifelines sample, 2.8% had CKD and 65% answered the health literacy questionnaire. Individuals who answered the questionnaire had fewer comorbidities. Participants with low health literacy were more likely to be older, and have a low socioeconomic status and higher BMI (Table 1).

**Table 1:** Comparison of baseline characteristics between CKD patients with low and adequate health literacy

Variables	Adequate health literacy (n = 1816)	Low health literacy (n = 926)	P value	n
Demographic characteristics				
Sex *% of females*	54.6	57.9	0.10^a^	2742
Age *mean, (SD)*	52.7 (15.7)	58.0 (15.4)	** < 0.001**^b^	2742
Education *%*				
Low	3.2	11.8	** < 0.001**^a^	2638
Intermediate	63.6	77.4		
High	33.1	10.9		
Monthly household income *%*				
< 1000 €	5.8	6.8	** < 0.001**^a^	2652
1000–3000 €	54.1	60.2		
> 3000 €	26.5	15.2		
Information not given	13.7	17.8		
Clinical characteristics				
Smoking *%*	18.2	19.8	0.30^a^	2716
BMI *median, (IQR)*	26.5 (5.9)	27.4 (5.4)	** < 0.001**^c^	2741
Number of disease domains* *%*				
0	14.4	7.9	** < 0.001**^a^	2742
1	21.6	14.8		
2	23.4	21.9		
3	21.2	26.6		
4 or more	19.4	28.8		
eGFR *median, (IQR)*	80.4 (42.0)	69.8 (40.6)	** < 0.001**^c^	2742
CKD stage *%*				
1	38.8	31.4	** < 0.001**^a^	2742
2	22.9	22.9		
3	37.4	44.4		
4 and 5	0.9	1.3		

*CKD* Chronic kidney disease, *BMI* body mass index, *eGFR* estimated glomerular filtration rate

P values < 0.05 marked in bold

*Comorbidities grouped into 11 disease domains according to organ system

^a^Method: Pearson’s chi-square test

^b^Method: independent samples *t* test

^c^Method: Mann–Whitney test

### Association between low health literacy and multimorbidity

**Fig. 1:**
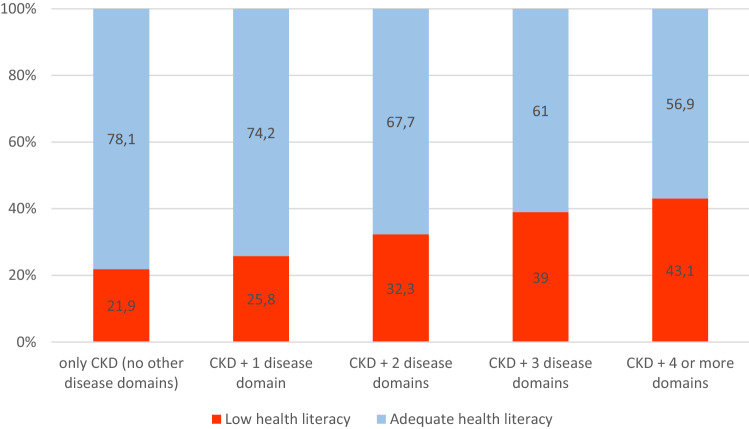
Share of CKD patients with low and adequate health literacy by number of disease domains

Patients with low health literacy had more diseases simultaneously with their CKD (Table 2 and Fig. 1). This trend remained the same after stratification by sex and age, and older patients presented with more diseases than younger ones (Supplementary Appendix II and III). The number of diseases presented by one patient ranged from 0 to 14, and the number of disease domains from 0 to 8. Having low health literacy increased the chance of having multiple disease domains, and this association was stronger for greater numbers of disease domains (Table 3).

**Table 2:** Comparison of baseline prevalence of single diseases and disease domains between CKD patients with low and adequate health literacy

Diseases	Total sample (n = 2742)^a^	Adequate health literacy (n = 1816)	Low health literacy (n = 926)	P value^b^
**Gastrointestinal diseases** *%*	61.3	57.8	68.4	** < 0.001**
Fatty liver disease *%*	60.3	56.8	67.2	** < 0.001**
Gastric disease *%*	2.9	2.0	4.5	** < 0.001**
Ulcerative colitis *%*	0.8	0.9	< 1.1	0.33
Crohn’s disease *%*	< 0.4	< 0.6	< 1.1	0.38
Celiac disease *%*	< 0.4	< 0.6	< 1.1	0.63
**Cardiovascular diseases** *%*	44.2	41.0	50.4	** < 0.001**
Hypertension *%*	43.2	40.0	49.5	** < 0.001**
Heart failure *%*	8.9	8.0	10.6	**0.02**
Vascular disease *%*	7.9	7.2	9.4	**0.04**
Atrial fibrillation *%*	3.4	3.0	4.3	0.07
Pacemaker *%*	0.8	0.7	1.1	0.32
Heart transplant *%*	< 0.4	< 0.6	< 1.1	0.99
**Endocrine diseases %**	38.6	34.3	47.1	** < 0.001**
Hypercholesterolemia *%*	31.8	28.5	38.3	** < 0.001**
Diabetes *%*	11.0	9.7	13.6	**0.002**
Hypothyroidism *%*	< 0.4	< 0.6	< 1.1	0.60
Hyperthyroidism *%*	0.4	< 0.6	< 1.1	0.56
**Pulmonary diseases** *%*	33.2	31.4	36.5	**0.008**
COPD *%*	28.2	26.7	31.1	**0.02**
Asthma *%*	8.2	7.7	9.3	0.16
**Central nervous system diseases** *%*	23.6	22.9	25.1	0.20
Migraine *%*	17.0	17.2	16.5	0.66
Back or neck hernia *%*	7.5	6.7	9.2	**0.02**
Epilepsy *%*	0.6	< 0.6	1.1	**0.01**
Multiple sclerosis *%*	< 0.4	< 0.6	< 1.1	0.77
**Ophthalmic** **and** **Otorhinolaryngologic diseases** *%*	10.8	9.3	13.8	** < 0.001**
Ophthalmic disease *%*	8.8	7.4	11.4	** < 0.001**
Otorhinolaryngologic disease *%*	2.5	2.1	3.1	0.12
**Psychiatric diseases** *%*	10.6	9.0	13.7	** < 0.001**
Anxiety disorder *%*	8.9	7.6	11.6	**0.001**
Depressive disorder *%*	3.5	2.4	5.5	** < 0.001**
**Musculoskeletal diseases** *%*	6.5	5.7	8.2	**0.01**
Rheumatoid arthritis *%*	3.3	2.9	4.1	0.08
Arthrosis *%*	2.3	1.8	3.2	**0.01**
Gout *%*	1.9	1.9	1.8	0.95
Osteoporosis *%*	< 0.4	< 0.6	< 1.1	0.09
**Hematologic diseases** *%*	3.0	3.0	3.0	0.94
Anemia *%*	1.3	1.3	1.4	0.77
Thrombotic disease *%*	1.6	1.7	1.6	0.95
Hemorrhagic disease *%*	< 0.4	< 0.6	< 1.1	0.63
**Genitourinary diseases** *%*	2.5	2.3	3.0	0.23
Benign prostatic hypertrophy *%*	2.2	1.9	2.7	0.16
Chronic bladder infection *%*	< 0.4	< 0.6	< 1.1	0.28
Double ovary extirpation *%*	< 0.4	< 0.6	< 1.1	0.23
**Dermatologic diseases** *%*	2.4	2.5	2.1	0.43
Eczema *%*	1.9	1.9	1.7	0.71
Psoriasis *%*	0.4	< 0.6	< 1.1	0.52
Severe acne *%*	< 0.4	< 0.6	< 1.1	0.31

Dementia and Parkinson’s disease not displayed: presented by fewer than 0.05% of patients

P values < 0.05 marked in bold

*COPD* Chronic obstructive pulmonary disease. Participants considered ‘affected’ in a disease domain (in bold) if positive for any disease listed below corresponding domain

^a^All cases complete

^b^Method: Pearson’s chi-square test

**Table 3:** Association between low health literacy and number of disease domains

Number of domains^a^		Crude model				Adjusted for age and sex				Adjusted for age, sex, and eGFR				Adjusted for age, sex, eGFR, smoking, and BMI		
		OR	95% CI	p		OR	95% CI	p		OR	95% CI	p		OR	95% CI	p
1		1.25	0.90–1.72	0.18		1.12	0.80–1.56	0.52		1.13	0.81–1.58	0.47		1.06	0.74–1.51	0.74
2		1.71	1.25–2.33	**0.001**		1.42	1.02–1.98	**0.04**		1.47	1.05–2.05	**0.02**		1.31	0.91–1.88	0.14
3		2.29	1.68–3.10	** < 0.001**		1.76	1.25–2.46	**0.001**		1.83	1.30–2.58	** < 0.001**		1.59	1.10–2.31	**0.01**
4 or more		2.71	2.00–3.68	** < 0.001**		1.92	1.36–2.71	** < 0.001**		2.00	1.41–2.83	** < 0.001**		1.70	1.16–2.49	**0.006**
n		2742				2742				2742				2715		

P values < 0.05 marked in bold

Comorbidities grouped into 11 disease domains according to organ system

^a^In all analyses, zero (zero comorbidities in addition to CKD) = reference category for number of domains; adequate health literacy = reference category for health literacy

### Multimorbidity patterns in CKD patients with low and adequate health literacy

**Fig. 2:**
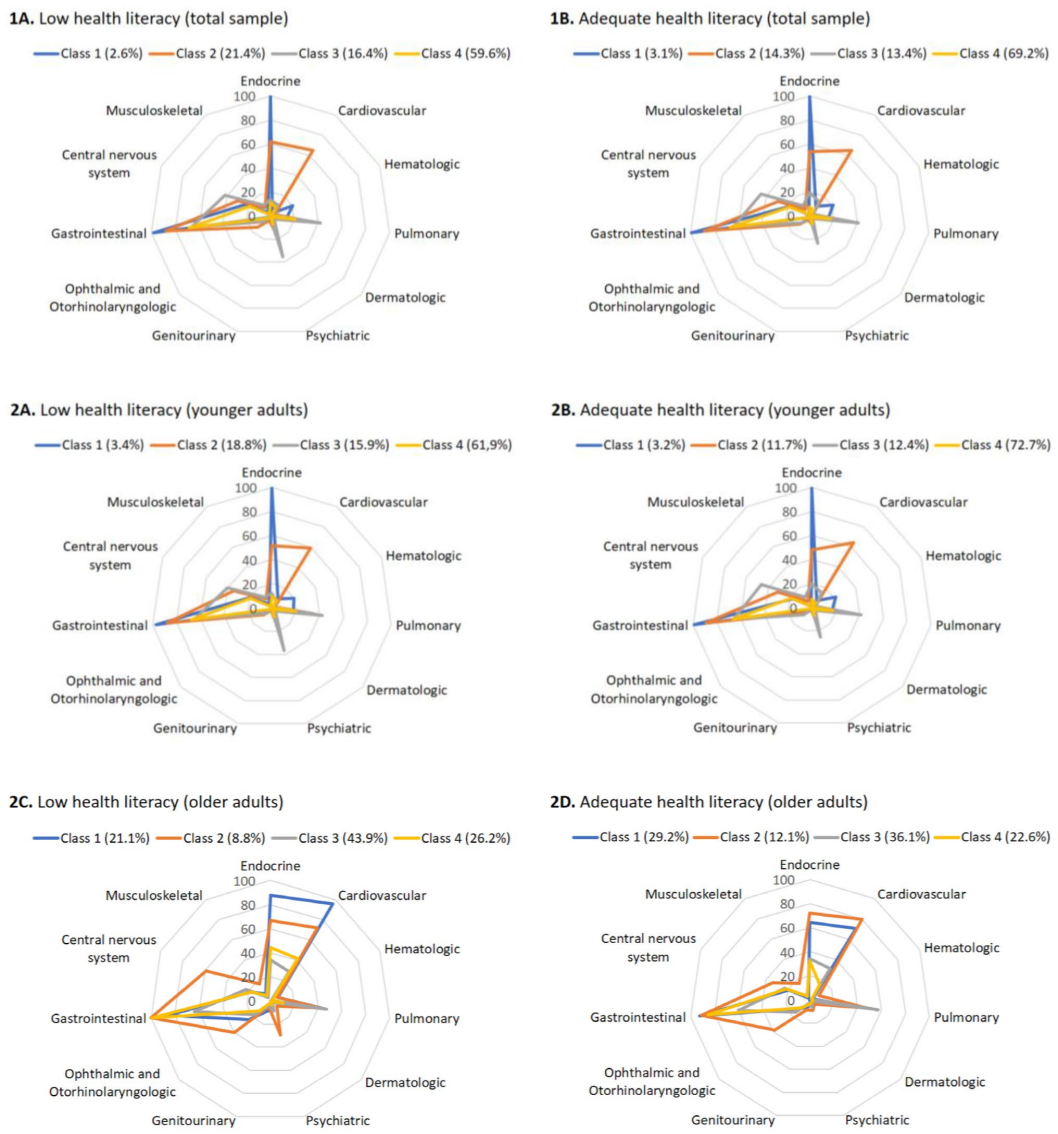
Patterns of multimorbidity for groups of low and adequate health literacy, in total sample (1A and 1B) and stratified by age (2A–2D)

The patterns of multimorbidity were very similar for both health literacy groups (Fig. 2, charts 1A and 1B). For both groups, the best-fitting model had four classes: (1) gastrointestinal and endocrine; (2) gastrointestinal, endocrine, cardiovascular, and pulmonary; (3) gastrointestinal, pulmonary, psychiatric, and neurologic; and (4) gastrointestinal. Supplementary Appendix IV shows the percentages of each disease domain per class. In the group with low health literacy, classes 1–4 represented 2.6%, 21.4%, 16.4%, and 59.6% of the sample, respectively. For the adequate health literacy groups, percentages were 3.1%, 14.3%, 13.4%, and 69.2%. Patterns were similar between health literacy groups across sex (Supplementary Appendix V). Patterns were also similar between health literacy groups among younger patients, but slightly different among older ones (Fig. 2, charts 2A–2D). Older patients with low health literacy had a relatively greater share of cardiovascular disease in class 4, and psychiatric and central nervous system diseases in class 2, when compared to older patients with adequate health literacy.

## Discussion

This study showed that CKD patients have a high prevalence of comorbidities. Patients with low health literacy, especially when older, were more likely to have an even higher number of comorbidities. Moreover, for both levels of health literacy, the patterns of multimorbidity were similar, in the total sample and by sex. For the subgroup of older patients, however, those with low health literacy had a higher prevalence of most comorbidities, with a relatively greater share of cardiovascular, psychiatric, and central nervous system diseases.

Low health literacy is a strong independent indicator of the number of comorbidities among CKD patients. The association between low health literacy and comorbidities showed a dose–response gradient and was independent of age, sex, lifestyle, and CKD severity. This finding reinforces the robustness of the results, and the association most likely stems from the less effective health behaviors and self-management skills of patients with low health literacy [2]. These patients, thus, develop more comorbidities, which may negatively impact the prognosis of CKD. Our results contribute to the body of literature on the association between low health literacy and multimorbidity, which has until now shown mixed results [23, 24]. Our study underlines the negative effects of low health literacy on multimorbidity, with a much larger sample and a more comprehensive set of comorbidities than in previous studies.

Interestingly, the patterns of multimorbidity were similar between patients with low and adequate health literacy in the total sample and when divided by sex, and only slightly different in older patients. These similar patterns indicate that, even though patients with low health literacy have more comorbidities than their adequate health literacy counterparts, the combination of comorbidities is roughly the same. This means that support of multimorbid CKD patients with low health literacy need not focus on different groups of diseases, but rather on general measures related to CKD care.

Among older adults, patients with low health literacy showed a higher prevalence of most comorbidities, which could be explained in two ways. First, patients with low health literacy have worse health behaviors and self-management skills, leading to aggravation of existing diseases and the development of new comorbidities, which accumulate over the life course [2]. A second explanation is that patients with low health literacy have poor access to care [25]. This could be due to a lack of resources to access care, or to a lack of knowledge to adequately use the available care. In either case, the result is a suboptimal use of medical resources for treating existing conditions and preventing new ones. The apparent differences in associations with health literacy by age suggest that the negative effects of low health literacy develop over time, being less in younger patients but more evident among older ones.

We found that patients with CKD had a high prevalence of specific comorbidities, which could overburden patients and negatively impact health outcomes. In the multimorbidity patterns studied, the most prevalent disease domains regarded gastrointestinal, endocrine, and cardiovascular diseases. These diseases are already known for co-occurring with CKD, given that they share various risk factors [26, 27]. The presence of multiple comorbidities increases the overall disease burden for patients [28]. Associated with this, it may lead to more polypharmacy with consequent poorer medication adherence, to increased psychological distress, and to a worse quality of life [1, 29]. Furthermore, the presence of these comorbidities could accelerate eGFR decrease, either because combining different treatments becomes more intricate for patients, or because gastrointestinal, endocrine, and cardiovascular disease might be in the causal pathway toward CKD [1, 19, 30].

Strengths of this study include its large population-based sample, with a wide selection of physical and mental chronic diseases, diagnosed by a combination of subjective and objective methods, using an internationally accepted coding system, and reported by age and sex. Furthermore, we employed latent class analysis, a sophisticated analysis technique to capture the heterogeneity in disease distribution. Latent class analysis is a person- rather than disease-oriented technique, providing results that meet current guidelines for patient-centered research and care.

Some limitations of our study should also be taken into account. First, we performed a cross-sectional analysis, which did not allow us to study whether multimorbidity patterns and their association with low health literacy change over time. Second, the health literacy questionnaire which we used is a self-report instrument focusing mainly on functional health literacy. This may have led to underestimations of low health literacy due to self-report, thereby reducing the power of our analysis. Nonetheless, our instrument is a validated tool that has been used in other studies [31]. Third, data on albuminuria were missing for 54% of our sample. However, this is unlikely to lead to important selection bias because albuminuria was assessed for a random subgroup of the Lifelines sample and its assessment stopped because of logistical reasons not related to CKD. Fourth, 65% of the participants answered the health literacy questionnaire. This reduced the power of our study, as the number of comorbidities was lower than among the participants that did not answer the questionnaire.

Public health practitioners and healthcare professionals should be aware of the importance of addressing low health literacy already at younger ages to prevent the development of multimorbidity during the life course. This aligns with current guidelines that focus more on prevention in healthcare services [32]. Moreover, the similar patterns of multimorbidity presented in both health literacy groups suggest that it is not necessary to change the focus of the care of low-health-literate patients regarding which groups of comorbidities should be prioritized. Care for them should be directed at the same comorbidities as those of their adequate-health-literate counterparts, but with extra support to overcome the challenges intrinsic to low health literacy. In clinical practice, this support could be achieved by using health-literacy-friendly strategies to improve self-management, health behaviors, and the quality and accessibility of CKD care. Patients could benefit from informational materials designed as narratives, such as photo stories, which are more recognizable, relevant, and engaging to patients [33, 34]. These materials could be used to promote the acquisition of health-related skills and CKD knowledge, and to increase patients’ self-efficacy and motivation. Moreover, strengthening social support by engaging family members or friends of the patient could enhance disease management at home. Clinicians could also be supported by strategies to facilitate decision-making with patients with low health literacy [35]. This could be achieved through training to help clinicians tailor their communication strategies to the specific needs of each patient.

Future research should study the factors that mediate the association between low health literacy and multimorbidity to support the development of interventions to assist CKD patients. Ideally, research should be performed longitudinally, noting the trajectory and evolution of comorbidities, as well as health literacy, along the life course.

### Conclusion

This study shows that, in a context of high prevalence of comorbidity, CKD patients with low health literacy are more likely to have a higher number of comorbidities than patients with adequate health literacy. Moreover, the multimorbidity patterns are similar for both groups of health literacy, differing slightly at older ages. This age difference suggests that the negative effects of low health literacy are more evident in  aging groups. Therefore, improving low health literacy could be an intervention targeted to decrease multimorbidity along the life course of CKD patients.

## Supplementary Information

Below is the link to the electronic supplementary material.

Supplementary file1 (PDF 1183 KB)

## Data Availability

The datasets analyzed during the current study are available in the Lifelines Biobank repository, at https://www.lifelines.nl/researcher.
