# Supplementary material for: Multimorbidity prevalence and patterns and their associations with health literacy among chronic kidney disease patients
Source: J Nephrol. 2022 Jan 5;35(6):1709–19. doi: 10.1007/s40620-021-01229-1 (PMC9300533; doi:10.1007/s40620-021-01229-1)
Supplement: Supplementary file 1 — Supplementary file1 (PDF 1183 KB) [file 40620_2021_1229_MOESM1_ESM.pdf]

Supplementary Information

Journal of Nephrology

Title: Multimorbidity prevalence and patterns and their associations with health literacy among chronic kidney disease patients

Gurgel do Amaral, MS[1]; Reijneveld, SA[1]; Meems, LMG[2]; Almansa, J[1]; Navis, GJ[3]; de Winter AF[1]

[1] University of Groningen, University Medical Center Groningen, Department of Health Sciences, Community and Occupational Medicine, The Netherlands
[2] University of Groningen, University Medical Center Groningen, Department of Cardiology, The Netherlands
[3] University of Groningen, University Medical Center Groningen, Department of Nephrology, The Netherlands

Corresponding author
MS Gurgel do Amaral
University Medical Center Groningen
Hanzeplein 1
Building 3217, room 617
Groningen, The Netherlands, 9713GZ
Email: m.silva.gurgel.do.amaral@umcg.nl
ORCID ID: 0000-0002-8830-4687

E-mail addresses of the authors
s.a.reijneveld@umcg.nl
ORCID ID: 0000-0002-1206-7523
l.m.g.meems@umcg.nl
ORCID ID: 0000-0001-8627-7139
j.almansa.ortiz@umcg.nl
ORCID ID: 0000-0001-9724-9328
g.j.navis@umcg.nl
a.f.de.winter@umcg.nl
ORCID ID: 0000-0002-9380-4889

Supplementary Appendix I

Classification criteria for single diseases and disease domains, using data from the Lifelines Cohort Study

GASTROINTESTINAL DISEASE

Fatty liver disease

- Non-alcoholic fatty liver disease (NAFLD) (XI-K75.8)
    - HSI>36 & no alcohol consumption
- Alcoholic fatty liver disease (AFLD) (XI-K70.0)
    - HSI>36 & alcohol consumption

  *NB: HSI= 8 * (ALT/AST) + BMI (+2 if female) (+2 if diabetes mellitus)*

Gastric disease

- Peptic ulcer (XI-K25)
    - Self-reported, **and**
    - use of H2-antagonists, prostaglandins, proton pump inhibitors (PPI)
- Symptoms of gastric disease (XVIII-R10/R11/R12/R13)
    - Self-reported complaints: heartburn, stomach pain, nausea, vomiting, reflux, **and**
    - use of H2-antagonists, prostaglandins, PPI

Ulcerative Colitis (XI-K51)

- Self-reported

Crohn's disease (XI-K50)

- Self-reported

Celiac Disease (XI-K90)

- Self-reported celiac disease, **and**
- gluten free diet

H2-antagonists, prostaglandins, PPI = ATC code A02B

Gastrointestinal disease if at least one of following present:

- Fatty liver disease
- Gastric disease
- Ulcerative colitis
- Crohn's disease
- Celiac disease

CARDIOVASCULAR DISEASE

Hypertension (IX-I10)

- BP> 140/90mmHG, **or**
- use of anti-hypertensive medication

Heart failure (IX-I50)

- Self-reported disease, **and**
- use of (any) HF-related medication (diuretics, ACE-I, aldosterone antagonist, beta-blocker, angiotensin receptor blocker)

Vascular disease (IX-I21, IX-I25.2, IX-I64)

- Self-reported MI, stroke, PCI & CABG, **and**
- use of vitamin K antagonists **or** ascal/acetylsalicylic acid **or** clopidogrel **or** use of (simva-) statin

Atrial fibrillation (IX-I48)

- Self-reported, 'diagnosed by MD' **and** use of vitamin K antagonists, **or**
- AF on ECG **and** use of vitamin K antagonists, **or**
- CHADVASC <2 **and** AF on ECG

Pacemaker (XXI-Z95.0)

- Self-reported

Heart transplant (XXI-Z94.1)

- Self-reported

Anti-hypertensive medication= ATC-codes C02, C03, C04, C07, C08, C09

Diuretics= ATC-codes C03A, C03B, C03C, C03E, C03X

Aldosterone antagonist= ATC-code C03D

Beta-blocker= ATC-code C07

Calcium antagonist= ATC-code C08

ACE-inhibitors and ANG II antagonists = ATC-code C09

Vitamin K antagonists = ATC-code B01AA

Ascal = ATC-code B01AC06

Acetylsalicylic acid= ATC-code B01AC08

Clopidogrel= ATC-code B01AC04

Statin= ATC-code C10AA

Simvastatin= ATC-code C10AA01

Cardiovascular disease if at least one of following present:

- Hypertension
- Heart failure
- Vascular disease
- Atrial fibrillation
- Pacemaker
- Heart transplant

ENDOCRINE DISEASE

Hypercholesterolemia (IV-E78.0)

- Self-reported myocardial infarction (HEALTH19) and cholesterol >=5.0, **or**
- use of lipid lowering medication, **or**
- cholesterol >= 6.5 mmol/L

Diabetes (IV-E10/E11/E12/E14)

- Self-reported diabetes (HEALTH17A, HEALTH17B), **or**
- use of oral anti-diabetics and/or insulin, **or**
- fasting glucose levels >6.99, **or**
- non-fasting glucose level >11.0

NB: we defined different types of diabetes, excluding diabetes related to pregnancy but not to any (other) medical cause.

Hypothyroidism (IV-E03)

- Use of thyroid hormones, **or**
- TSH>4 & FT4<11.0, **or**
- TSH>10

Hyperthyroidism (IV-E05)

- TSH <0.5 & FT4>19.5

Oral anti-diabetics= ATC-code A10B

Insulin= ATC-code A10A

Thyroid hormones= ATC-code H03

Lipid lowering drugs= ATC-codes C10A, C10B

Endocrine disease if at least one of following present:

- Hypercholesterolemia
- Diabetes
- Hypothyroidism
- Hyperthyroidism

PULMONARY DISEASE

COPD (X-J44)

NB: for diagnosis of COPD we included only individuals aged >40 years.

- If spirometry data is available, COPD is present in case of:
    - COPD_GOLD classification: I-IV, **or**
- if spirometry data is not available, COPD is present in case of:
    - Self-reported COPD & use of asthma/COPD medication & age >40, **or**
    - phlegm production during day or night, or immediately after waking up during at least 3 months per year (*Chronic mucus hypersecretion (X-J42)*), **or**
    - coughing/phlegm production during the day or night, or immediately after waking up during at least 3 months per year (*Chronic bronchitis (X-J40)*)

Asthma (X-J45)

- Doctors diagnosed asthma, **or**
- 2 or more self-reported symptoms of: wheezing, attacks during rest, being awakened by an

attack **and** use of asthma /COPD medication, **or**
- self-reported asthma **and** use of asthma/COPD medication

Asthma/COPD medication = ATC-codes R03AC, R03CC, R03AK, R03BA, R03BB, R03BC01, R03BC03, R03DA04, R03DC

Pulmonary disease if at least one of following present:
- Asthma
- COPD

CENTRAL NERVOUS SYSTEM DISEASE

Migraine (VI-G43)

- Self-reported migraine

Back or neck hernia (XIII-51.2)

- Self-reported back or neck hernia

Epilepsy (VI-G40)

- Self-reported epilepsy **and** use of anti-epileptics

Multiple Sclerosis (VI-G35)

- Self-reported multiple sclerosis

Dementia (V-F00/F01/F02/F03)
- Self-reported dementia, **or**
- MMSE-score <24 if education level low/medium, **or**
- MMSE-score <25 if education level high

Parkinson's disease (VI-G20)

- Self-reported Parkinson's disease **and** use of anti-Parkinson's drugs

Anti-epileptics = ATC-code N03

Anti-Parkinson's drugs= ATC-codes N04BC02, N04BB01, N04BC07, N04BX02, N04BA02, N04BC05, N04BD02, N04BC04, N04BC09, N04BD01, N04BX01

Central nervous system disease if at least one of following present:
- Migraine
- Back or neck hernia
- Epilepsy
- Multiple sclerosis
- Dementia
- Parkinson's disease

OPHTALMIC AND OTORHINOLARYNGOLOGIC DISEASE

Ophthalmic disease
- Cataract (VII-H25/H26), **or**
- severe visual impairment (VII-H54.1)

Otorhinolaryngologic disease

- Chronic sinusitis of throat and/or nose (X-J32) **and** use of local or systemic decongestive drugs, **or**
- severe hearing impairment (VIII-H90)

Decongestive drugs= ATC-code R01

Ophthalmic and otorhinolaryngologic disease if at least one of following present:
- Ophthalmic disease
- Otorhinolaryngologic disease

PSYCHIATRIC DISEASE

NB: Presence of (symptoms of) psychiatric diseases based on outcomes from MINI interview

Anxiety disorder (V-F40-F41)

- Any anxiety disorder

Depressive disorder (V-F32.0-33.0) and dysthymic disorder
- Current depressive disorder, **or**
- dysthymic disorder **and** use of antidepressants

Antidepressants= ATC-code N06A

Psychiatric disease if at least one of following present:
- Current anxiety disorder
- Current depressive disorder

MUSCULOSKELETAL DISEASE

Rheumatoid Arthritis (XIII-M06.9)

- Self-reported rheumatoid arthritis **and** use of NSAIDS/DMARDS/ TNF-alpha inhibitors/ IL inhibitors, **or**
- self-reported rheumatoid arthritis **and** use of B- and T-cell inhibitors **and/or** methotrexate, **or**
- 2 or more self-reported symptoms of pain in hands & feet, or joint stiffness, **and** use of NSAIDS/DMARDS/ TNF-alpha inhibitor/ IL inhibitors, **or**
- 2 or more self-reported symptoms of pain in hands & feet, or joint stiffness, **and** use of B- and T-cell inhibitors **and/or** methotrexate

Arthrosis (XIII-M19.9)
- Self-reported arthrosis **and** use of analgesics (paracetamol, NSAIDs, opioids), **or**
- self-reported symptoms of pain in hands (HEALTH51) & feet **and** use of analgesics (paracetamol, NSAIDs, opioids), **or**
- self-reported symptoms of joint stiffness **and** use of analgesics (paracetamol, NSAIDs, opioids)

Gout (XIII-M10)

- Use of anti-gout medication

Osteoporosis (XIII-M81)

- Self-reported osteoporosis **and** use of calci-chew D3 **and** bisphosphonates

Opioids= ATC-code N02A

Paracetamol= ATC-code N02BE01

NSAIDs= ATC-codes M01AE, M01AB

DMARDS = ATC- code M01

TNF-alpha inhibitor= ATC-code L04AB

IL inhibitors= ATC-code L04AC

B- and T-cell inhibitors= ATC-codes LO4AA24, L01XC02

Methotrexate= ATC-code L01BA01

Anti-gout medication: ATC-code M04

Musculoskeletal disease if at least one of following present:

- Rheumatoid arthritis
- Arthrosis
- Gout
- Osteoporosis

<u>HEMATOLOGIC DISEASE</u>

<u>Anemia (III-D50 untill D64)</u>

- Self-reported anemia **<u>and</u>** use of iron supplementation, **<u>or</u>**
- Hb <6.0 if female, **<u>or</u>** -
- Hb<6.5 if male.

<u>Thrombotic disease</u>

- Self-reported thrombosis (IX-I82) **<u>and</u>** use of anti-thrombotic drugs, **<u>or</u>**
- self-reported lung emboli (IX-I26) **<u>and</u>** use of anti-thrombotic drugs, **<u>or</u>**
- self-reported coagulation disorder (III-D68.9) **<u>and</u>** use of anti-thrombotic drugs, **<u>or</u>**
- thrombocytosis (III-D47.3).
    - Thrombocyte count >750*10E9/L.

<u>Hemorrhagic disease</u>

- Self-reported coagulation disorder (III-D68.9) **<u>and</u>** use of anti-hemorrhagic drugs, **<u>or</u>**
- thrombocytopenia (III-D69.3/D69.4/D69.4/D69.5/D69.6)
    - Thrombocyte count <60*10E9/L

Iron supplementation= ATC-code B03

Anti-thrombotic drugs= ATC-codes B01AA, B01AB

Anti-hemorrhagic drugs= ATC-code B02

Hematologic disease if at least one of following present:

- Anemia
- Thrombotic disease
- Hemorrhagic disease

GENITOURINARY DISEASE

Benign prostatic hypertrophy (BPH) (XIV-N40)

- Use of BPH-related drugs

Chronic bladder infection (XIV-N39)

- Self-reported chronic bladder infection (HEALTH72D2) **and** use of anti- microbial drugs

Double ovary extirpation (XXI-Z90.7)

- Self-reported surgical extirpation of 2 ovaries (FEM7C1) **and** use of female hormones

Anti-microbial drugs= ATC-codes G01, J01

BPH-related drugs= ATC-code G04C

Female hormones= ATC-code G03C

Genitourinary disease if at least one of following present:

- Benign prostatic hypertrophy
- Chronic bladder infection
- Double ovary extirpation

DERMATOLOGIC DISEASE

Eczema (XII-L30.9)

- Self-reported eczema (HEALTH2) **and** use of emollients and/or dermatological corticosteroids

Psoriasis (XII-L40.9)

- Self-reported psoriasis (HEALTH72H3) **and** psoriasis suppressor

Severe acne (XII-L70.9)

- Self-reported severe acne (HEALTH72H1) **and** use acne suppressor

Acne suppressor= ATC-code D10

Emollients= ATC-code D02

Psoriasis suppressor= ATC-code D05

Dermatologic disease if at least one of following present:

- Eczema
- Psoriasis
- Severe acne

Supplementary Appendix II

Comparison of baseline prevalence of single diseases and disease domains, stratified by sex and age

Part A. Analyses stratified by sex

Supplementary Table 1: Comparison of baseline prevalence of single diseases and disease domains among female CKD patients with low and adequate health literacy*

**Diseases**	**Total sample** (n=1,528)[a]	**Adequate health literacy** (n=992)	**Low health literacy** (n=536)	**P value**[b]
**Gastrointestinal disease** %	58.0	53.1	67.2	**<0.001**
Fatty liver disease %	57.1	52.2	66.0	**<0.001**
Gastric disease %	2.4	1.2	4.7	**<0.001**
Ulcerative colitis %	0.7	<1.1	<1.9	0.32
Crohn's disease %	<0.7	<1.1	<1.9	0.48
Celiac disease %	<0.7	<1.1	<1.9	0.66
**Cardiovascular disease** %	38.4	34.9	45.0	**<0.001**
Hypertension %	37.7	34.2	44.2	**<0.001**
Heart failure %	8.6	8.1	9.7	0.28
Vascular disease %	4.3	4.2	4.5	0.82
Atrium fibrillation %	2.3	2.3	2.2	0.92
Pacemaker %	0.7	<1.1	<1.9	0.93
Heart transplant %	<0.7	<1.1	0.0	0.46
**Endocrine disease** %	36.2	30.9	45.9	**<0.001**
Hypercholesterolemia %	29.1	25.0	36.6	**<0.001**
Diabetes %	8.4	6.8	11.4	**0.002**
Hypothyroidism %	<0.7	<1.1	<1.9	0.93
Hyperthyroidism %	<0.7	<1.1	<1.9	0.20
**Pulmonary disease** %	29.6	27.5	33.4	**0.02**
COPD %	24.1	22.9	26.3	0.14
Asthma %	9.0	7.6	11.6	**0.009**
**Central nervous system disease** %	29.7	28.6	31.7	0.21
Migraine %	23.9	23.6	24.4	0.71
Back or neck hernia %	7.1	6.3	8.6	0.09
Epilepsy %	<0.7	<1.1	<1.9	0.10
Multiple sclerosis %	<0.7	<1.1	<1.9	0.48
**Ophthalmic and Otorhinolaryngologic disease** %	10.9	8.6	15.1	**<0.001**
Ophthalmic disease %	9.0	6.9	12.9	**<0.001**
Otorhinolaryngologic disease %	2.3	1.9	3.0	0.18
**Psychiatric disease** %	12.1	9.8	16.4	**<0.001**
Anxiety disorder %	10.3	8.2	14.2	**<0.001**
Depressive disorder %	4.0	2.8	6.2	**0.001**
**Musculoskeletal disease** %	6.1	4.6	8.8	**0.001**
Rheumatoid arthritis %	3.7	3.1	4.9	0.09
Arthrosis %	2.6	2.2	3.4	0.18
Gout %	<0.7	<1.1	<1.9	**0.04**[c]

Osteoporosis *%*	<0.7	<1.1	<1.9	0.10
**Hematologic disease** *%*	3.1	3.4	2.4	0.30
Anemia *%*	1.8	2.0	<1.9	0.32
Thrombotic disease *%*	1.2	1.4	<1.9	0.42
Hemorrhagic disease *%*	<0.7	0.0	<1.9	0.17
**Genitourinary disease** *%*	<0.7	<1.1	<1.9	0.44
Benign prostatic hypertrophy *%*	NA	NA	NA	NA
Chronic bladder infection *%*	<0.7	<1.1	<1.9	0.95
Double ovary extirpation *%*	<0.7	<1.1	<1.9	0.25
**Dermatologic disease** *%*	2.4	2.4	2.2	0.82
Eczema *%*	2.0	1.9	2.1	0.85
Psoriasis *%*	<0.7	<1.1	<1.9	0.48
Severe acne *%*	<0.7	<1.1	0.0	0.46

COPD: chronic obstructive pulmonary disease. NA: not applicable. Dementia and Parkinson's disease not displayed: presented by fewer than 0.1% patients
** Participants considered 'affected' in a disease domain (in bold) if positive for any disease listed below corresponding domain; [a] All cases complete; [b] Method: Pearson's chi-square test, [c] Prevalence higher among CKD patients with low health literacy*

Supplementary Table 2: Comparison of baseline prevalence of single diseases and disease domains among male CKD patients with low and adequate health literacy*

**Diseases**	**Total sample** (n=1,214)[a]	**Adequate health literacy** (n=824)	**Low health literacy** (n=390)	**P value**[b]
**Gastrointestinal disease** %	65.5	63.3	70.0	**0.02**
Fatty liver disease %	64.3	62.3	68.7	**0.03**
Gastric disease %	3.5	3.0	4.4	0.24
Ulcerative colitis %	0.9	<1.3	<2.6	0.73
Crohn's disease %	<0.9	<1.3	0.0	0.49
Celiac disease %	0.0	0.0	0.0	NA
**Cardiovascular disease** %	51.5	48.4	57.9	**0.002**
Hypertension %	50.2	47.1	56.7	**0.002**
Heart failure %	9.1	7.9	11.8	**0.03**
Vascular disease %	12.4	10.7	16.2	**0.007**
Atrium fibrillation %	4.9	3.8	7.2	**0.01**
Pacemaker %	1.0	<1.3	<2.6	0.18
Heart transplant %	<0.9	<1.3	<2.6	0.59
**Endocrine disease** %	41.7	38.3	48.7	**0.001**
Hypercholesterolemia %	35.3	32.6	40.8	**0.006**
Diabetes %	14.3	13.2	16.7	0.11
Hypothyroidism %	<0.9	<1.3	0.0	0.33
Hyperthyroidism %	<0.9	<1.3	0.0	0.23
**Pulmonary disease** %	37.6	36.2	40.8	0.12
COPD %	33.4	31.3	37.7	**0.03**
Asthma %	7.3	7.9	6.2	0.28
**Central nervous system disease** %	15.9	15.9	15.9	1.00
Migraine %	8.2	9.5	5.6	**0.02**
Back or neck hernia %	8.2	7.3	10.0	0.11
Epilepsy %	<0.9	<1.3	<2.6	0.06
Multiple sclerosis %	<0.9	<1.3	<2.6	0.59
**Ophthalmic and Otorhinolaryngologic disease** %	10.8	10.2	12.1	0.33
Ophthalmic disease %	8.5	8.0	9.5	0.39
Otorhinolaryngologic disease %	2.7	2.4	3.3	0.36
**Psychiatric disease** %	8.7	8.1	10.0	0.28
Anxiety disorder %	7.2	6.9	7.9	0.52
Depressive disorder %	2.8	1.9	4.6	**0.008**
**Musculoskeletal disease** %	7.1	6.9	7.4	0.74
Rheumatoid arthritis %	2.7	2.5	3.1	0.60
Arthrosis %	1.8	<1.3	3.1	**0.02**
Gout %	3.6	3.9	3.1	0.48
Osteoporosis %	0.0	0.0	0.0	NA
**Hematologic disease** %	2.9	2.4	3.8	0.17
Anemia %	<0.9	<1.3	<2.6	**0.03**[c]
Thrombotic disease %	2.1	1.9	2.6	0.48
Hemorrhagic disease %	<0.9	<1.3	0.0	0.49
**Genitourinary disease** %	5.2	4.6	6.4	0.19
Benign prostatic hypertrophy %	4.9	4.1	6.4	0.08
Chronic bladder infection %	<0.9	<1.3	0.0	0.17

Double ovary extirpation *%*	NA	NA	NA	NA
**Dermatologic disease** *%*	2.4	2.7	<2.6	0.35
Eczema *%*	1.7	1.9	<2.6	0.41
Psoriasis *%*	<0.9	<1.3	<2.6	0.84
Severe acne *%*	<0.9	<1.3	0.0	0.49

COPD: chronic obstructive pulmonary disease. NA: not applicable. Dementia and Parkinson's disease not displayed: presented by zero patients

** Participants considered 'affected' in disease domain (in bold) if positive for any disease listed below corresponding domain; [a] All cases complete; [b] Method: Pearson's chi-square test, [c] Prevalence higher among CKD patients with low health literacy*

Part B. Analyses stratified by age

Supplementary Table 3: Comparison of baseline prevalence of single diseases and disease domains among CKD patients younger than 65, with low and adequate health literacy*

**Diseases**	**Total sample** (n=1,888)[a]	**Adequate health literacy** (n=1,318)	**Low health literacy** (n=570)	**P value**[b]
**Gastrointestinal disease** %	53.8	51.1	60.0	**<0.001**
Fatty liver disease %	52.6	50.0	58.6	**0.001**
Gastric disease %	2.3	1.7	3.9	**0.004**
Ulcerative colitis %	0.8	1.0	<1.8	0.32
Crohn's disease %	<0.6	<0.8	0.0	0.14
Celiac disease %	<0.6	<0.8	0.0	0.51
**Cardiovascular disease** %	33.5	31.2	38.8	**0.001**
Hypertension %	32.5	30.3	37.5	**0.002**
Heart failure %	5.5	5.1	6.3	0.28
Vascular disease %	3.8	3.6	4.2	0.50
Atrium fibrillation %	2.0	1.8	2.3	0.51
Pacemaker %	<0.6	<0.8	<1.8	0.63
Heart transplant %	<0.6	<0.8	0.0	0.51
**Endocrine disease** %	30.0	27.1	36.7	**<0.001**
Hypercholesterolemia %	24.1	21.5	30.0	**<0.001**
Diabetes %	7.8	7.1	9.3	0.11
Hypothyroidism %	<0.6	<0.8	<1.8	0.93
Hyperthyroidism %	<0.6	<0.8	<1.8	0.75
**Pulmonary disease** %	30.6	28.3	36.0	**0.001**
COPD %	25.3	23.3	30.0	**0.002**
Asthma %	8.5	8.0	9.8	0.18
**Central nervous system disease** %	24.3	22.6	28.1	**0.01**
Migraine %	18.2	17.6	19.5	0.33
Back or neck hernia %	7.2	6.0	9.8	**0.003**
Epilepsy %	0.7	<0.8	<1.8	**0.002**[c]
Multiple sclerosis %	<0.6	<0.8	<1.8	0.62
**Ophthalmic and Otorhinolaryngologic disease** %	5.0	4.8	5.6	0.45
Ophthalmic disease %	3.2	3.0	3.9	0.31
Otorhinolaryngologic disease %	2.1	2.0	2.3	0.67
**Psychiatric disease** %	12.2	10.1	17.2	**<0.001**
Anxiety disorder %	10.3	8.5	14.6	**<0.001**
Depressive disorder %	4.2	2.8	7.4	**<0.001**
**Musculoskeletal disease** %	5.9	5.2	7.5	**0.04**
Rheumatoid arthritis %	3.8	3.1	5.3	**0.02**
Arthrosis %	2.0	1.7	2.6	0.21
Gout %	1.3	1.4	<1.8	0.58
Osteoporosis %	0.0	0.0	0.0	NA
**Hematologic disease** %	2.4	2.4	2.5	0.89
Anemia %	1.4	1.4	<1.8	0.71
Thrombotic disease %	1.0	0.9	<1.8	0.53
Hemorrhagic disease %	0.0	0.0	0.0	NA

**Genitourinary disease** *%*	1.2	1.2	<1.8	0.76
Benign prostatic hypertrophy *%*	0.8	0.8	<1.8	0.79
Chronic bladder infection *%*	<0.6	<0.8	0.0	0.14
Double ovary extirpation *%*	<0.6	<0.8	<1.8	0.54
**Dermatologic disease** *%*	2.4	2.4	2.3	0.85
Eczema *%*	1.9	1.7	2.3	0.43
Psoriasis *%*	<0.6	<0.8	0.0	0.08
Severe acne *%*	<0.6	<0.8	0.0	0.35

COPD: chronic obstructive pulmonary disease. NA: not applicable. Dementia and Parkinson's disease not displayed: presented by fewer than 0.05% of patients
** Participants considered 'affected' in a disease domain (in bold) if positive for any disease listed below corresponding domain; [a] All cases complete; [b] Method: Pearson's chi-square test, [c] Prevalence higher among CKD patients with low health literacy*

Supplementary Table 4: Comparison of baseline prevalence of single diseases and disease domains among CKD patients older than 65, with low and adequate health literacy*

**Diseases**	**Total sample** (n=854)[a]	**Adequate health literacy** (n=498)	**Low health literacy** (n=356)	**P value**[b]
**Gastrointestinal disease** %	78.0	75.3	81.7	**0.03**
Fatty liver disease %	77.3	74.7	80.9	**0.03**
Gastric disease %	4.1	3.0	5.6	0.06
Ulcerative colitis %	<1.2	<2.1	<2.9	0.94
Crohn's disease %	<1.2	0.0	<2.9	0.24
Celiac disease %	<1.2	0.0	<2.9	0.24
**Cardiovascular disease** %	67.9	67.1	69.1	0.53
Hypertension %	66.9	65.7	68.5	0.38
Heart failure %	16.4	15.7	17.4	0.50
Vascular disease %	17.1	16.7	17.7	0.69
Atrium fibrillation %	6.7	6.0	7.6	0.37
Pacemaker %	2.1	<2.1	<2.9	0.81
Heart transplant %	<1.2	<2.1	<2.9	0.81
**Endocrine disease** %	57.7	53.4	63.8	**0.003**
Hypercholesterolemia %	48.8	46.8	51.7	0.16
Diabetes %	18.1	16.5	20.5	0.13
Hypothyroidism %	<1.2	<2.1	0.0	0.40
Hyperthyroidism %	<1.2	<2.1	<2.9	0.18
**Pulmonary disease** %	38.8	39.8	37.4	0.48
COPD %	34.5	35.7	32.9	0.38
Asthma %	7.6	7.0	8.4	0.45
**Central nervous system disease** %	22.1	23.5	20.2	0.26
Migraine %	14.3	16.1	11.8	0.08
Back or neck hernia %	8.4	8.6	8.1	0.80
Epilepsy %	<1.2	<2.1	<2.9	0.77
Multiple sclerosis %	<1.2	<2.1	<2.9	0.81
**Ophthalmic and Otorhinolaryngologic disease** %	23.7	21.3	27.0	0.05
Ophthalmic disease %	21.0	19.1	23.6	0.11
Otorhinolaryngologic disease %	3.4	2.6	4.5	0.13
**Psychiatric disease** %	7.0	6.2	8.1	0.28
Anxiety disorder %	5.9	5.2	6.7	0.35
Depressive disorder %	1.9	<2.1	<2.9	0.23
**Musculoskeletal disease** %	8.0	7.0	9.3	0.23
Rheumatoid arthritis %	2.2	2.2	<2.9	0.97
Arthrosis %	2.8	<2.1	4.2	**0.04**
Gout %	3.2	3.2	3.1	0.92
Osteoporosis %	<1.2	<2.1	<2.9	0.21
**Hematologic disease** %	4.3	4.6	3.9	0.63
Anemia %	1.2	<2.1	<2.9	0.24
Thrombotic disease %	3.0	3.6	<2.9	0.25
Hemorrhagic disease %	<1.2	<2.1	<2.9	0.81
**Genitourinary disease** %	5.5	5.0	6.2	0.46
Benign prostatic hypertrophy %	5.2	4.8	5.6	0.60
Chronic bladder infection %	<1.2	<2.1	<2.9	0.81

Double ovary extirpation *%*	<1.2	0.0	<2.9	0.24
**Dermatologic disease** *%*	2.3	2.8	<2.9	0.28
Eczema *%*	1.8	2.4	<2.9	0.09
Psoriasis *%*	<1.2	<2.1	<2.9	0.40
Severe acne *%*	<1.2	0.0	0.0	NA

COPD: chronic obstructive pulmonary disease. NA: not applicable. Dementia and Parkinson's disease not displayed: presented by fewer than 0.1% of patients

** Participants considered 'affected' in a disease domain (in bold) if positive for any disease listed below corresponding domain; [a] All cases complete; [b] Method: Pearson's chi-square test*

Supplementary Appendix III

Association between low health literacy and number of disease domains, stratified by sex and age

Part A. Analyses stratified by sex

Supplementary Table 5: Association between low health literacy and number of disease domains in female CKD population*

Number of	Crude model			Adjusted for age			Adjusted for age and eGFR			Adjusted for age, eGFR, smoking, and BMI		
domains[a]	OR	95% CI	p	OR	95% CI	p	OR	95% CI	p	OR	95% CI	p
1	1.39	0.93-2.07	0.11	1.20	0.80-1.82	0.38	1.23	0.81-1.86	0.33	1.07	0.69-1.66	0.76
2	1.88	1.27-2.77	**0.002**	1.40	0.92-2.14	0.12	1.45	0.95-2.22	**0.08**	1.18	0.74-1.86	0.49
3	2.52	1.71-3.73	**<0.001**	1.73	1.13-2.68	**0.01**	1.82	1.18-2.81	**0.007**	1.47	0.91-2.36	0.11
4 or more	3.23	2.21-4.73	**<0.001**	2.05	1.33-3.18	**0.001**	2.15	1.39-3.34	**0.001**	1.71	1.05-2.78	**0.03**
n	1,528			1,528			1,528			1,511		

** Comorbidities grouped into 11 disease domains according to organ system*
[a] In all analyses, zero (i.e., zero comorbidities in addition to CKD) = reference category for number of domains; adequate health literacy = reference category for health literacy
Models on right nested in models to their left

Supplementary Table 6: Association between low health literacy and number of disease domains in male CKD population*

Number of	Crude model			Adjusted for age			Adjusted for age and eGFR			Adjusted for age, eGFR, smoking, and BMI		
domains[a]	OR	95% CI	p	OR	95% CI	p	OR	95% CI	p	OR	95% CI	p
1	1.04	0.60-1.81	0.89	0.98	0.55-1.73	0.94	0.98	0.56-1.74	0.95	1.09	0.58-2.02	0.80
2	1.50	0.89-2.52	0.12	1.37	0.79-2.36	0.26	1.42	0.82-2.46	0.21	1.53	0.84-2.80	0.17
3	2.03	1.22-3.37	**0.006**	1.71	0.98-2.97	0.06	1.78	1.02-3.09	**0.04**	1.82	0.98-3.36	0.06
4 or more	2.12	1.27-3.56	**0.004**	1.70	0.96-3.01	0.07	1.77	1.00-3.14	0.05	1.75	0.93-3.31	0.08
n	1,214			1,214			1,214			1,204		

** Comorbidities grouped into 11 disease domains according to organ system*
[a] In all analyses, zero (i.e., zero comorbidities in addition to CKD) = reference category for number of domains; adequate health literacy= reference category for health literacy
Models on right nested in models to their left

Part B. Analyses stratified by age

Supplementary Table 7: Association between low health literacy and number of disease domains in CKD population younger than 65*

Number of domains[a]	Crude model			Adjusted for age and sex			Adjusted for age, sex, and eGFR			Adjusted for age, sex, eGFR, smoking, and BMI		
	OR	95% CI	p	OR	95% CI	p	OR	95% CI	p	OR	95% CI	p
1	1.12	0.80-1.58	0.50	1.08	0.76-1.53	0.68	1.09	0.76-1.55	0.64	1.03	0.71-1.49	0.88
2	1.60	1.15-2.23	**0.005**	1.45	1.02-2.05	**0.04**	1.48	1.04-2.11	**0.03**	1.33	0.91-1.94	0.15
3	2.10	1.49-2.96	**<0.001**	1.86	1.28-2.68	**0.001**	1.91	1.32-2.76	**0.001**	1.68	1.12-2.51	**0.01**
4 or more	2.49	1.75-3.52	**<0.001**	2.03	1.38-2.99	**<0.001**	2.09	1.42-3.07	**<0.001**	1.77	1.16-2.72	**0.009**
n	1,888			1,888			1,888			1,867		

** Comorbidities grouped into 11 disease domains according to organ system*

[a] In all analyses, zero (i.e., zero comorbidities in addition to CKD) = reference category for number of domains; adequate health literacy = reference category for health literacy

Models on right nested in models to their left.

Supplementary Table 8: Association between low health literacy and number of disease domains in CKD population older than 65*

Number of domains[a]	Crude model			Adjusted for age and sex			Adjusted for age, sex, and eGFR			Adjusted for age, sex, eGFR, smoking, and BMI		
	OR	95% CI	p	OR	95% CI	p	OR	95% CI	p	OR	95% CI	p
1	1.38	0.33-5.73	0.66	1.23	0.29-5.18	0.77	1.49	0.35-6.44	0.59	1.38	0.31-6.18	0.67
2	1.38	0.34-5.52	0.65	1.26	0.31-5.14	0.74	1.58	0.38-6.61	0.53	1.39	0.32-6.05	0.66
3	1.68	0.43-6.63	0.46	1.54	0.38-6.15	0.54	1.93	0.47-7.98	0.36	1.65	0.39-7.08	0.50
4 or more	1.96	0.50-7.71	0.34	1.62	0.41-6.47	0.50	2.05	0.50-8.45	0.32	1.75	0.41-7.51	0.46
n	854			854			854			848		

** Comorbidities grouped into 11 disease domains according to organ system*

[a] In all analyses, zero (zero comorbidities in addition to CKD) = reference category for number of domains; adequate health literacy = reference category for health literacy

Models on right nested in models to their left

Supplementary Appendix IV

Percentage of each disease domain per class in latent class analysis

Part A. Tables with results of latent class analysis for CKD patients with low health literacy.

Supplementary Table 9: Percentage of disease domains per latent class in total CKD sample with low health literacy*

**Disease domain**[a]	**Class 1** (%)	**Class 2** (%)	**Class 3** (%)	**Class 4** (%)
Endocrine	100	61,58	13,8	10,83
Cardiovascular	2,5	65,12	10,82	8,32
Hematologic	19,96	3,8	2,07	0,7
Pulmonary	13,61	35,94	42,09	20,8
Dermatologic	1,1	1,47	3,87	1,37
Psychiatric	16,63	14,3	35,58	7,25
Genitourinary	0,83	3,75	0,8	0,48
Ophthalmic and Otorhinolaryngologic	1,85	14,39	7,45	2,38
Gastrointestinal	98,29	88,05	66,85	68,5
Central nervous system	23,37	30,05	41,83	19,42
Musculoskeletal	3,55	8,48	11	1,87

** Result from latent class analysis using disease domains as observed variables*

[a] *Comorbidities grouped into 11 disease domains according to organ system*

Supplementary Table 10: Percentage of disease domains per latent class in female CKD patients with low health literacy*

**Disease domain**[a]	**Class 1** (%)	**Class 2** (%)	**Class 3** (%)	**Class 4** (%)
Endocrine	96,91	59,78	12,35	11,12
Cardiovascular	12,12	60,55	9,89	8,07
Hematologic	21,05	4,11	2,46	0,98
Pulmonary	13,72	35,21	38,5	18,65
Dermatologic	0,15	1,63	3,4	1,5
Psychiatric	14,26	17,72	37,36	7,81
Genitourinary	0,95	0,98	0,4	0,22
Ophthalmic and Otorhinolaryngologic	3,2	16,37	5,79	2,58
Gastrointestinal	97,63	88,46	65,2	67,04
Central nervous system	24,35	36,16	45,75	22,78
Musculoskeletal	3,71	9,3	10,67	2,16

** Result from latent class analysis using disease domains as observed variables*

[a] *Comorbidities grouped into 11 disease domains according to organ system*

Supplementary Table 11: Percentage of disease domain per latent class in male CKD patients with low health literacy*

Disease domain[a]	Class 1 (%)	Class 2 (%)	Class 3 (%)	Class 4 (%)
Endocrine	100	64,24	16,99	10,77
Cardiovascular	0	73,13	14,28	8,93
Hematologic	17,56	3,01	0,49	0,37
Pulmonary	15,06	36,77	56,02	21,81
Dermatologic	4,08	1,13	5,18	1,01
Psychiatric	18,66	11,01	25,07	6,64
Genitourinary	1,92	7,27	3,3	0,69
Ophthalmic and Otorhinolaryngologic	0	12	11,25	1,89
Gastrointestinal	96,79	88,05	68,97	70,92
Central nervous system	16,37	23,91	27,89	14,86
Musculoskeletal	4,32	7,84	8,93	1,71

** Result from latent class analysis using disease domains as observed variables*

[a] *Comorbidities grouped into 11 disease domains according to organ system*

Supplementary Table 12: Percentage of disease domains per latent class in CKD patients below 65 with low health literacy*

Disease domain[a]	Class 1 (%)	Class 2 (%)	Class 3 (%)	Class 4 (%)
Endocrine	100	51,35	12,36	9,76
Cardiovascular	9,28	59,03	6,36	7,2
Hematologic	20,06	2,81	2,31	0,69
Pulmonary	17,77	34,3	42,51	19,99
Dermatologic	1,14	1,62	3,84	1,44
Psychiatric	16,53	17,82	36,79	7,27
Genitourinary	0,87	2,13	0,6	0,39
Ophthalmic and Otorhinolaryngologic	2,68	8,61	6,96	1,84
Gastrointestinal	97,22	87,77	65,34	67,87
Central nervous system	20,36	34,72	40,54	19,47
Musculoskeletal	3,97	9,04	11,1	1,77

** Result from latent class analysis using disease domains as observed variables*

[a] *Comorbidities grouped into 11 disease domains according to organ system*

Supplementary Table 13: Percentage of disease domains per latent class in CKD patients above 65 with low health literacy*

**Disease domain**[a]	**Class 1** (%)	**Class 2** (%)	**Class 3** (%)	**Class 4** (%)
Endocrine	87,66	66,94	34	44,16
Cardiovascular	95,97	71,85	28,82	41,48
Hematologic	6,85	6,49	0,64	1,78
Pulmonary	42,64	41,98	47,03	10,52
Dermatologic	0	6,83	1,89	0
Psychiatric	7,59	29,5	8,92	3,21
Genitourinary	8,5	5,15	4,56	2,44
Ophthalmic and Otorhinolaryngologic	23,55	40,01	17,76	12,66
Gastrointestinal	85,33	100	63,69	99,2
Central nervous system	18,49	59,16	22,6	17,44
Musculoskeletal	7,14	16,82	3,19	5,5

** Result from latent class analysis using disease domains as observed variables*

[a] *Comorbidities grouped into 11 disease domains according to organ system*

Part B. Tables with results of latent class analysis for CKD patients with adequate health literacy

Supplementary Table 14: Percentage of disease domains per latent class in total CKD sample with adequate health literacy*

**Disease domain**[a]	**Class 1** (%)	**Class 2** (%)	**Class 3** (%)	**Class 4** (%)
Endocrine	100	53,67	19,26	7,52
Cardiovascular	9,23	64,59	14,35	5,85
Hematologic	21,1	2,75	3,11	0,87
Pulmonary	15,7	30,03	40,8	17,38
Dermatologic	1,33	1,68	3,54	2,05
Psychiatric	6,36	8,18	23,81	6,69
Genitourinary	0,38	3,64	0,95	0,39
Ophthalmic and Otorhinolaryngologic	1,66	10,77	9,06	1,61
Gastrointestinal	99,48	88,44	64,37	66,87
Central nervous system	18,62	28,75	44,45	18,4
Musculoskeletal	0,92	6,4	10,1	1,53

** Result from latent class analysis using disease domains as observed variables*

[a] *Comorbidities grouped into 11 disease domains according to organ system*

Supplementary Table 15: Percentage of disease domains per latent class in female CKD patients with adequate health literacy*

**Disease domain**[a]	**Class 1** (%)	**Class 2** (%)	**Class 3** (%)	**Class 4** (%)
Endocrine	100	54,36	20,77	7,61
Cardiovascular	5,81	57,81	18,89	5,12
Hematologic	28,91	3,47	3,85	1,35
Pulmonary	16,46	26,7	45,82	15,93
Dermatologic	1,17	1,36	4,52	1,97
Psychiatric	7,91	9,39	27,69	9
Genitourinary	0	0,84	0,89	0,19
Ophthalmic and Otorhinolaryngologic	1,53	11,46	10,87	1,74
Gastrointestinal	99,99	88,6	65,57	65,18
Central nervous system	22,36	35,99	49,92	22,99
Musculoskeletal	0,55	6,98	12,18	1,88

** Result from latent class analysis using disease domains as observed variables*

[a] *Comorbidities grouped into 11 disease domains according to organ system*

Supplementary Table 16: Percentage of disease domains per latent class in male CKD patients with adequate health literacy*

**Disease domain**[a]	**Class 1** (%)	**Class 2** (%)	**Class 3** (%)	**Class 4** (%)
Endocrine	100	50,77	12,82	5,83
Cardiovascular	22,3	63,45	5,91	8,34
Hematologic	11,48	2,19	0,58	0,34
Pulmonary	12,26	34	44,76	11,44
Dermatologic	1,36	1,96	3,32	1,74
Psychiatric	4,01	8,09	10,4	3,55
Genitourinary	0,99	6,35	1,27	0,49
Ophthalmic and Otorhinolaryngologic	0,67	10,27	5,6	0,63
Gastrointestinal	96,36	86,96	61,29	71,73
Central nervous system	10,63	24,75	19,36	13,25
Musculoskeletal	1,01	6,47	2,98	1,65

** Result from latent class analysis using disease domains as observed variables*

[a] *Comorbidities grouped into 11 disease domains according to organ system*

Supplementary Table 17: Percentage of disease domains per latent class in CKD patients below 65 with adequate health literacy*

**Disease domain**[a]	**Class 1** (%)	**Class 2** (%)	**Class 3** (%)	**Class 4** (%)
Endocrine	100	47,93	19,8	7,08
Cardiovascular	6,8	64,32	14,75	5,08
Hematologic	21,75	2,36	3,26	0,89
Pulmonary	16,91	26,48	41,46	17,41
Dermatologic	1,45	1,53	3,73	2,07
Psychiatric	6,78	9,26	25,44	6,92
Genitourinary	0,24	2,11	1,01	0,32
Ophthalmic and Otorhinolaryngologic	1,7	6,27	9,15	1,51
Gastrointestinal	98,89	89,06	65,94	66,37
Central nervous system	17,9	31,08	46,12	18,54
Musculoskeletal	1,35	6,15	10,82	1,58

** Result from latent class analysis using disease domains as observed variables*

[a] *Comorbidities grouped into 11 disease domains according to organ system*

Supplementary Table 18: Percentage of disease domains per latent class in CKD patients above 65 with adequate health literacy*

**Disease domain**[a]	**Class 1** (%)	**Class 2** (%)	**Class 3** (%)	**Class 4** (%)
Endocrine	64,45	72,23	34,39	32,16
Cardiovascular	70,69	79,8	31,05	15,43
Hematologic	2,57	8,57	1,37	1,72
Pulmonary	22,37	47,48	57,1	2,41
Dermatologic	0	5,39	2,01	2,11
Psychiatric	3,71	9,2	6,59	2,82
Genitourinary	6,62	8,78	3,15	4,42
Ophthalmic and Otorhinolaryngologic	10,73	38,62	15,83	10,2
Gastrointestinal	92,47	90,77	59,81	85,28
Central nervous system	19,45	33,74	23,21	21,68
Musculoskeletal	1,75	16,55	3,88	3,61

** Result from latent class analysis using disease domains as observed variables*

[a] *Comorbidities grouped into 11 disease domains according to organ system*

Supplementary Appendix V

Patterns of multimorbidity for groups of low and adequate health literacy, stratified by sex

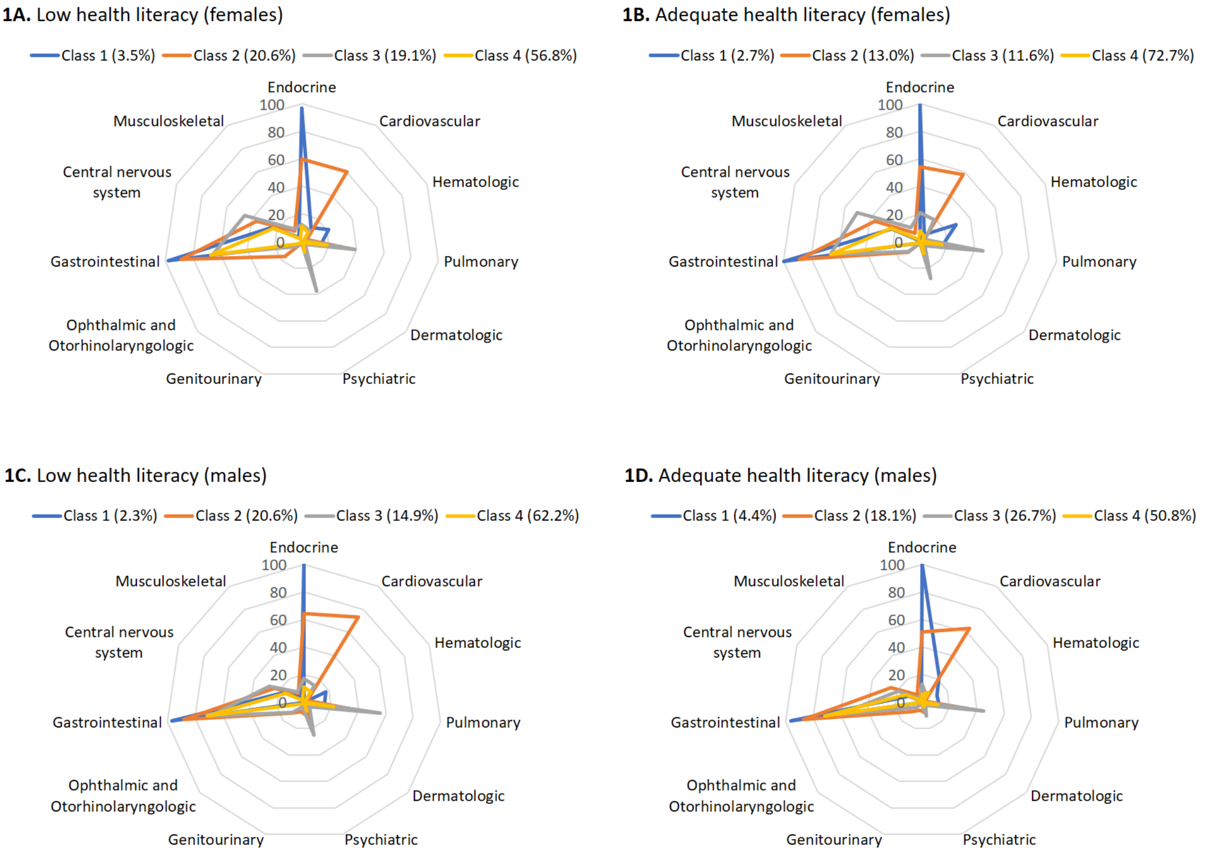

Suppl. Fig. 1 Patterns of multimorbidity for groups of low and adequate health literacy, stratified by sex
